# Costs of Immunization Programs for 10 Vaccines in 94 Low- and Middle-Income Countries From 2011 to 2030

**DOI:** 10.1016/j.jval.2020.07.010

**Published:** 2021-01

**Authors:** So Yoon Sim, Elizabeth Watts, Dagna Constenla, Shuoning Huang, Logan Brenzel, Bryan N. Patenaude

**Affiliations:** 1 International Vaccine Access Center, Johns Hopkins Bloomberg School of Public Health, Baltimore, MD, USA; 2 Department of International Health, Johns Hopkins Bloomberg School of Public Health, Baltimore, MD, USA; 3 GlaxoSmithKline Plc., Panama City, Panama; 4 Bill & Melinda Gates Foundation, Seattle, WA, USA

**Keywords:** health financing, investment, low- and middle-income countries

## Abstract

**Objectives:**

Understanding the level of investment needed for the 2021-2030 decade is important as the global community faces the next strategic period for vaccines and immunization programs. To assist with this goal, we estimated the aggregate costs of immunization programs for ten vaccines in 94 low- and middle-income countries from 2011 to 2030.

**Method:**

We calculated vaccine, immunization delivery and stockpile costs for 94 low- and middle-income countries leveraging the latest available data sources. We conducted scenario analyses to vary assumptions about the relationship between delivery cost and coverage as well as vaccine prices for fully self-financing countries.

**Results:**

The total aggregate cost of immunization programs in 94 countries for 10 vaccines from 2011 to 2030 is $70.8 billion (confidence interval: $56.6-$93.3) under the base case scenario and $84.1 billion ($72.8-$102.7) under an incremental delivery cost scenario, with an increasing trend over two decades. The relative proportion of vaccine and delivery costs for pneumococcal conjugate, human papillomavirus, and rotavirus vaccines increase as more countries introduce these vaccines. Nine countries in accelerated transition phase bear the highest burden of the costs in the next decade, and uncertainty with vaccine prices for the 17 fully self-financing countries could lead to total costs that are 1.3-13.1 times higher than the base case scenario.

**Conclusion:**

Resource mobilization efforts at the global and country levels will be needed to reach the level of investment needed for the coming decade. Global-level initiatives and targeted strategies for transitioning countries will help ensure the sustainability of immunization programs.

## Highlights

• Costing exercises have been an important pillar of global immunization agenda setting in the past decade, providing information on the level of investment needed to achieve the strategic goals set by the global immunization community.
• This study estimated the costs of immunization programs for 10 vaccines in 94 low- and middle-income countries from 2011 to 2030.
• The study leverages the latest available data on model parameters, updates the methodology to derive delivery cost per dose across 94 countries, and explores various scenarios that are relevant to strategic priorities in the future decade.
• As the global community works toward establishing a new vision and strategy, understanding and estimating the level of investment needed for the future decade is critical to decision making and priority setting for resource mobilization and allocation.
• The results of the study will provide insights that will help the global health community understand the future financing needs under various scenarios and prioritize health investments in low- and middle-income countries.

## Introduction

As the global community faces the next strategic period for vaccines and immunization programs, understanding the level of investment needed for the 2021 to 2030 decade is critical to mobilizing resources and developing effective strategies to increase access to vaccines around the world.

Costing exercises have been an important pillar of global immunization agenda setting. Wolfson et al (2018) presented the methods and results of the exercise undertaken by World Health Organization (WHO) and the United Nations Children’s Fund (UNICEF) for the Global Immunization Vision and Strategy (2006-2015), the first 10-year strategic framework.^1^ Gandhi et al (2013), members of the Decade of Vaccine Costing and Funding Technical Working Group, provided costing and financing projections for the Global Vaccine Action Plan (GVAP) (2011-2020) to describe investments needed to achieve the goals outlined in the collective roadmaps for enabling universal access to vaccines.^2^ Lydon et al (2014) explored further routine immunization delivery costs from 2011 to 2020.^3^ Portnoy et al (2015) estimated the resource needs for 94 countries to meet the GVAP goal at an aggregate level,^4^ and the results from the study were used to estimate the global return on investment for 1 dollar invested in immunization programs against 10 pathogens.^5^

Looking ahead to the coming decade, we estimated the costs of immunization programs for 10 vaccines in 94 low- and middle-income countries from 2011 to 2030 to inform Immunization Agenda 2030, a new global vision and strategy based on the lessons learned from the past decade.^6^ Although this study follows the tradition of the previous costing exercises, it contributes further to the literature by updating the methodologies, leveraging newly available data sources, and varying assumptions that are relevant to future strategic priorities.

## Methods

### Scope of the Analysis

The study focused on 94 low- and middle-income countries, including 73 countries supported by Gavi, the Vaccine Alliance, an international organization that brings together public and private sectors to increase access to new and underused vaccines through market-shaping efforts and cost-sharing for vaccines.^7^ The list of countries was based on the GVAP and Gavi’s country eligibility policy.^4^^,^^8^ These 94 low- and middle-income countries (LMICs) represented 50.3% of the total world population in 2018,^9^ and of these countries, 34 are classified as low income, 46 as lower-middle income, and 14 as upper-middle income countries.^10^ The average life expectancy at birth is 66.9 years, compared with 80.6 years for 36 OECD countries.^9^ Classification of the countries by WHO regions,^11^ country income level,^10^ and Gavi eligibility and country transition^8^ is available in Appendix 1 (in Supplemental Materials found at https://doi.org/10.1016/j.jval.2020.07.009).

The scope of the study consists of 10 vaccines—pentavalent (Penta), human papillomavirus (HPV), Japanese encephalitis (JE), measles (Measles), measles-rubella (MR), measles-mumps-rubella (MMR), meningococcal group A conjugate (MenA), pneumococcal conjugate (PCV), rotavirus (RV), and yellow fever (YF)—that are delivered through routine immunization or supplementary immunization activities (SIA) (Appendix 2 in Supplemental Materials found at https://doi.org/10.1016/j.jval.2020.07.009).

Immunization program costs for routine immunization and SIA are divided into 2 components: vaccine cost and immunization delivery cost. Vaccine cost includes the costs to procure vaccines, including costs of injection supplies and freight (shipment) per dose. Immunization delivery cost refers to nonvaccine costs to deliver immunization to target populations, including personnel costs such as salaries, per diem, and travel allowances that relate to labor function; cold chain equipment and maintenance and overhead related to the storage function; and vehicles, transport, and fuel costs related to transportation function. Immunization delivery cost also includes other capital costs and recurrent costs (Appendix 3 in Supplemental Materials found at https://doi.org/10.1016/j.jval.2020.07.009). The suggested categorization of immunization program costs into vaccine and immunization delivery costs differentiates the price of vaccines as commodities (vaccine price per dose) from the “price” of other health-systems–related components of immunization programs (delivery cost per dose), which is multiplied by the projected number of doses as the common quantity measure. It is also consistent with the existing literature and practices.^12^

The study was conducted from the perspective of the global immunization community, estimating the total aggregate costs borne by all funders, including the government, Gavi, the Vaccine Alliance, and other development partners. The study does not include household costs such as transportation or lost productive time due to immunization sessions.

### Vaccine Costs

Vaccine costs were estimated by multiplying the number of doses by the price per dose for each vaccine, country, and year.

$$Vaccine\;costs_{ijk}=\sum_{k=2011}^{2030}\sum_{j=1}^{94}\sum_{i=1}^{10}\left(number\;of\;doses_{ijk}\;\times \;price\;per\;dose_{ijk}\right)$$

The number of doses delivered is a function of the size of the target population, vaccine coverage rate, number of recommended doses for a fully immunized person, vaccine-specific wastage rate, and buffer stock rate.

$$Number\;of\;doses_{ijk}=Target\;population_{ijk}\times \;Coverage\;rate_{ijk}\times Number\;of\;recommended\;doses_{ij}\;\times \left(1+Wastage\;rate_{ij}\right)\times \left(1+Buffer\;stock\;rate_{i}\right)$$

For routine vaccines, the Vaccine Impact Modelling Consortium (VIMC) secretariat, which standardizes and coordinates the work of vaccine impact modeling research groups, provided the demographic data based on the United Nations World Population Prospects 2017 Revision^13^ as well as coverage data for 10 antigens based on WHO/UNICEF Estimates of National Immunization Coverage and Gavi’s operational forecast version 16 published in 2017.^14^ Gavi’s operational forecast projects the introduction years for new vaccines and assumes an increase in the coverage of each vaccine, reaching the coverage rate of diphtheria-tetanus-pertussis (DTP3) immunization within 2 or 3 years of introduction followed by a 1% increase in coverage every year up until 90% or 95%.^15^ For SIA vaccines, we used target population and coverage data provided by the VIMC secretariat.^14^ Vaccine-specific wastage rates are based on the simple average across Gavi-supported products for each corresponding vaccine outlined in Gavi’s Detailed Product Profile.^16^ Rates of 25% for routine immunization and 0% for SIA were used as buffer stock rates for all vaccines.^17^

Gavi provided historical weighted average prices of vaccines (2011-2017) and forecasted prices (2018-2030) for 73 countries,^18^ from which we used the simple average price across products for each vaccine. The Pan American Health Organization Revolving Fund price list provided historical vaccine prices (2011-2018) for 4 countries,^19^ and we obtained historical vaccine prices (2011-2021) for the remaining 17 countries from the UNICEF vaccine price list (Appendix 1 in Supplemental Materials found at https://doi.org/10.1016/j.jval.2020.07.009).^20^ We used the simple average of the historical prices of products for each vaccine. Due to uncertainty regarding future vaccine prices, we kept the price from the final year of available data constant throughout the remaining years, without indexing or correction for price increase or decrease, in accordance with Gavi’s price forecast. Instead, we varied the percent change in vaccine price for a probabilistic sensitivity analysis to account for potential price change in future years and determine uncertainty ranges. Gavi’s data on freight and supplies costs including syringe, recon syringe, and safety box were applied to all 94 countries.^18^

### Immunization Delivery Costs

The number of doses estimated for vaccines were multiplied by immunization delivery cost per dose for each vaccine, country, and year to estimate the total immunization delivery costs.

$$Immunization\;delivery\;costs_{ijk}=\sum_{k=2011}^{2030}\sum_{j=1}^{94}\sum_{i=1}^{10}\left(number\;of\;doses_{ijk}\times \;delivery\;cost\;per\;dose_{ij}\right)$$

For routine immunization, we extracted relevant data for baseline years, ranging from 2004 to 2019, from 89 Comprehensive Multi-Year Plans (cMYP) costing tools from 65 countries.^21^ In addition, we included 135 cost estimates for routine immunization in 19 low- and middle-income countries from the Immunization Delivery Cost Catalogue (IDCC)’s Excel-based database.^12^ Peer-reviewed articles, reports, and gray literature included in the IDCC were published between January 2005 and January 2018.^12^

We took multiple steps to prepare a combined dataset and estimate country-specific cost per dose estimates. We first standardized the definition of individual cost components by matching comparable components from cMYP and IDCC (Appendix 3 in Supplemental Materials found at https://doi.org/10.1016/j.jval.2020.07.009). For cMYP, we divided the total expenditure from the baseline years by the total number of doses to calculate cost per dose estimates that are comparable to estimates from the IDCC.

For countries with data from the cMYP or IDCC, we used the average of all available cost per dose estimates for the specific country from the combined dataset. For 18 countries without data, we conducted multiple linear regression analysis to predict the cost per dose for each country based on country characteristics. Cost per dose estimates that were used as dependent variables were log-transformed to account for the right-skewed distribution of the cost data. We identified seven predictors from published cost determinant studies^22^, ^23^, ^24^ that describe facility-level characteristics generalizable to the population level. Twelve additional country characteristics were identified as predictors and used as independent variables (Appendix 4).

To account for the difference between cMYP and IDCC data, we regressed cost per dose on 19 independent variables using 3 datasets: (1) the combined dataset, (2) the dataset that contains cMYP costing tools only, and (3) the dataset that contains IDCC cost estimates only. For each dataset, Akaike’s information criteria and Bayesian information criteria were examined to select the final model from each dataset through stepwise variable selection. Robust and clustered standard errors were used to account for multiple time periods for a single country.

Out-of-sample validation was then conducted to determine which of the 3 models performs best for the combined dataset. K-fold cross-validation (k = 10) was used to test each model for the 3 different datasets mentioned earlier. The final model from the combined dataset with the lowest average root mean square error (1.072) was used to predict the immunization cost per dose (Appendix 5 in Supplemental Materials found at https://doi.org/10.1016/j.jval.2020.07.009). Appendix 6 in Supplemental Materials found at https://doi.org/10.1016/j.jval.2020.07.009 shows the results of the multiple linear regressions used for prediction with and without dummy variables for cMYP (1 = cMYP, 0 = IDCC), confirming that there is no statistical difference between cMYP and IDCC values for predicting cost per dose conditional on other covariates. Duan’s smearing estimator was included to correct for retransformation bias.

$$Delivery\;cost\;per\;dose_{j}=Exp\left[\begin{array}{l} \beta_{0}+\beta_{1}\times \;Urban\;population\;+\;\beta_{2}\times \;Total\;number\;of\;births\;+\;\beta_{3}\;\times \;GDP\;growth\;+\\ \beta_{4}\;\times \;DTP3\;\mathrm{cov}erage\;rate\;+\;\beta_{5}\;\times \;GDP\;per\;capita\;+\;\beta_{6}\times Land\;area+\beta_{7}\;\times \;\\ Population\;growth\;+\;\beta_{8}\;\times \;Total\;number\;of\;doses\;delivered\;+\;\beta_{9}\;\times \\ Maternal\;mortality\;ratio\;+\;\beta_{10}\;\times \;Total\;number\;of\;DTP3\;doses\;delivered\;+\;\beta_{11}\;\times \;\\ Population\;density\;+\sum j\frac{e_{j}}{N} \end{array}\right]$$

Four countries (Marshall Islands, West Bank and Gaza, Micronesia, and Cape Verde) did not have enough information for predictors, so we applied the average imputed cost per dose from the combined dataset to these countries. Table 1 shows the summary statistics of the country-specific cost per dose estimates.

**Table 1:** Summary table for immunization delivery cost per dose estimates (USD 2018).

Category	Type	n‡	Average (SD)	Median	Range
Routine immunization∗	Total immunization delivery cost per dose	94	2.47 (1.96)	2.30	0.18-11.31
	Incremental cost per dose for introducing HPV§	42	3.90 (3.30)	2.86	0.52-13.44
	Incremental cost per dose for introducing PCV	21	1.20 (1.00)	1.06	0.15-3.50
	Incremental cost per dose for introducing Rotavirus vaccine	12	1.04 (0.64)	0.85	0.10-2.31
SIA†	Measles	17	0.95 (0.88)	0.70	0.04-3.63
	MR/MMR	13	0.88 (0.20)	0.84	0.69-1.46
	JE	2	0.69(0.01)	0.69	0.68-0.70
	MenA	15	0.51(0.39)	0.65	0.00-1.44
	Yellow Fever	4	0.65 (0.19)	0.69	0.42-0.81
	HPV SIA (Multi-age cohort)	1	0.53	0.53	0.53-0.53

∗ Ten vaccines for routine immunization: Pentavalent, human papilloma virus, Japanese encephalitis, measles, measles-rubella, measles-mumps-rubella, meningococcal group A conjugate, pneumococcal conjugate, rotavirus, and yellow fever vaccines.

† Seven vaccines for SIA: Human papilloma virus, Japanese encephalitis, measles, measles-rubella, measles-mumps-rubella, meningococcal group A conjugate, and yellow fever vaccines.

‡ Number of estimates in the model.

§ No distinction was made to HPV cost estimates from routine delivery via health facility and school delivery given uncertainty about country decisions regarding delivery strategies.

As the IDCC includes startup and introduction costs for newer vaccines, we calculated the mean incremental cost estimates for HPV, PCV, and RV vaccines and applied them to introduction years only. Given limited data available for other vaccines, startup and introduction costs were applied only to these 3 vaccines.

Immunization delivery costs for supplementary immunization activities (SIA), often referred to as operational costs, consist of nonvaccine costs to deliver immunizations of a more targeted and time-limited nature than routine immunization.^25^ Based on the data provided by the VIMC secretariat, catch-up, follow-up, or preventive campaigns for measles, MR, MenA, JE, and YF for eligible countries were included in the model. The multiple age cohort (girls of age 10-14) for HPV was categorized as SIA because it is optional for countries that choose to immunize additional girls beyond the routine cohort. Given supply constraints expected in the coming years, doses from the multi-age cohort were excluded for 2021-2025.

SIA-related information from the cMYP costing tools suffered from missing data and data inconsistency because SIA are irregular in frequency and SIA years often do not align with the cMYP baseline years. Considering the data quality issues with cMYP, we utilized information from the IDCC and additional data sources such as a systematic review by Gandhi et al 2013^25^ and budgeted amount per dose estimates from country proposals submitted to Gavi.^26^ We collected 52 estimates from three sources and calculated average cost per dose for each vaccine type (Table 1). These estimates were then applied to all 94 countries.

The costs of emergency vaccine stockpiles for meningitis and yellow fever have been included in this analysis. From 2011-2018, we used the historical disbursement data on emergency outbreak support and other strategic investments including stockpile^27^ and forecasted data on stockpile costs for future years (2019-2030).^28^

### Sensitivity Analysis

We conducted probabilistic sensitivity analysis using Monte Carlo simulations to determine uncertainty ranges for each scenario. We varied 5 parameters simultaneously and performed 10,000 model runs to construct 95% confidence intervals for total immunization program costs. We used the distribution of the cost per dose estimates from the compiled data mentioned earlier for 3 parameters—country-specific routine immunization delivery cost per dose; vaccine-specific SIA delivery cost per dose; and incremental delivery cost per dose for PCV, HPV, and RV vaccines. A uniform distribution was used for the percent change in vaccine price and stockpile cost per year (between ±15%).

### Scenario Analysis for Routine Immunization Delivery Cost

A scenario analysis for routine immunization delivery cost was conducted to vary the assumption about the relationship between delivery cost and coverage rate. In addition to the base case scenario, where we used constant delivery cost per dose, we incorporated an additional scenario in which delivery cost per dose incrementally increases with coverage rate by using the cost function presented in Ozawa et al 2018^29^ (Appendix 7 in Supplemental Materials found at https://doi.org/10.1016/j.jval.2020.07.009). Although previous systematic reviews^29^, ^30^, ^31^ examined the evidence on incremental cost and effect of specific interventions that increased immunization coverage rate, there is still limited evidence on interaction between incremental costs to deliver immunization to harder-to-reach populations, economies of scale, and health systems efficiency gains when the interventions are scaled up to the national level. In addition, 71% of the data used for the cost function are from upper-middle-income or high-income countries.^29^ Given these limitations, we presented the results using constant delivery cost per dose as the primary results and those from the scenario analysis as an upper-bound estimate.

### Scenario Analysis for Fully Self-Financing Countries

Gavi’s policy requires individual countries to co-finance all Gavi-supported routine vaccines, and the requirement differs across 4 transition classifications—initial self-financing phase, preparatory transition phase, accelerated transition phase, and fully self-financing phase—based on the eligibility and transition policy.^32^ In 2018, 17 countries were in fully self-financing phase, and 9 countries were in accelerated transition phase (Appendix 1 in Supplemental Materials found at https://doi.org/10.1016/j.jval.2020.07.009). To support these transitioning countries to sustain immunization programs and introduction of newer vaccines, some manufacturers have committed to offer fixed prices comparable to those paid by Gavi for vaccines for a limited time period and under specific conditions.^33^ As these commitments are not legally binding, we tested various scenarios in which countries will no longer have access to Gavi prices. From the WHO Market Information for Access to Vaccines database,^34^ we obtained the price information reported between 2011 and 2018 for countries that used procurement mechanisms other than UNICEF or Pan American Health Organization revolving fund defined as “other,” “self-procurement,” “other pool procurement,” and “sub-regional pool procurement.” We generated cost estimates for 17 fully self-financing countries from 2018 to 2020, using the minimum, maximum, and average price for each vaccine type. Because vaccine price tends to decrease with purchasing volume, these prices are calculated for 3 volume categories (low, medium, and high) defined based on the interquartile range of annual number of doses for each vaccine captured in the database. More detailed methodology can be found in Appendix 8 (in Supplemental Materials found at http://doi.org/10.1016/j.jval.2020.07.009).

## Results

**Figure 1:**
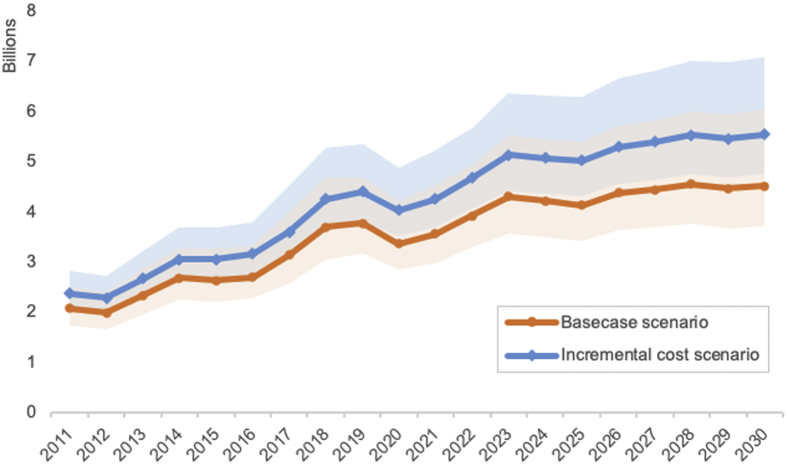
Total immunization program costs for 10 vaccines over time (base case scenario vs incremental delivery cost scenario).

The total costs of immunization programs for 10 vaccines in 94 countries from 2011 to 2030 is $70.8 billion (confidence interval [CI]: $56.6–$93.3), which averages approximately $35.8 (CI: $28.6-$47.2) per surviving infant, with an increasing trend over 2 decades. Given that the total income of these countries was approximately $75.2 trillion from 2011 to 2020, the total costs during this period ($28.4 billion) represent 0.04% of GDP. Vaccine costs account for 52.9% (60.9 %–43.2%) of the total costs, $37.4 billion ($34.4-$40.3), and immunization delivery costs represent 46.5% (38.4%-56.3%), $32.9 billion ($21.7-$52.5). Stockpile costs amount to $0.4 billion ($0.4-$0.5), 0.6% (0.5%-0.6%) of the total cost. When categorized by strategy, 87.4% of the total costs are attributed to routine immunization, and 12.0% of the costs are attributed to supplementary immunization activities (SIA) (Table 2). Under the scenario where delivery costs increase with coverage rate, the total costs of immunization programs are $84.1 billion ($72.8-$102.7) (Fig. 1). The total delivery costs from 2011 to 2030 is $46.3 billion ($35.2–$66.1), 40.6% higher than the delivery costs under the base case scenario.

**Table 2:** Cost of immunization programs for 10 vaccines by decade, 94 low- and middle-income countries (base case scenario).

	2011-2020	2021-2030	Total
By strategy			
Routine, total	$22.9 billion (18.4-29.5)	$39.0 billion (30.7-53.3)	$61.9 billion (49.2-82.3)
*Vaccine*	$12.4 billion (11.3-13.5)	$21.3 billion (19.5-23.0)	$33.7 billion (30.9-36.3)
*Delivery*	$10.5 billion (7.1-16.0)	$17.7 billion (11.2-30.4)	$28.2 billion (18.2-46.0)
SIA, total	$5.2 billion (3.3-9.5)	$3.2 billion (2.3-5.0)	$8.5 billion (5.7-14.5)
*Vaccine*	$2.2 billion (2.0-2.4)	$1.6 billion (1.5-1.7)	$3.8 billion (3.5-4.0)
*Delivery*	$3.1 billion (1.3-7.2)	$1.6 billion (0.9-3.3)	$4.7 billion (2.2-10.5)
Stockpile, total	$0.20 billion (0.17-0.24)	$0.22 billion (0.19-0.25)	$0.42 billion (0.36-0.49)
By component			
Immunization program, total	28.4 billion (22.7-36.3)	$42.4 billion (33.8-57.1)	$70.8 billion (56.5-93.4)
*Vaccine, total*	$14.6 billion (13.4-15.8)	$22.8 billion (21.1-24.60)	$37.4 billion (34.4-40.3)
*Delivery, total*	$13.6 billion (9.2-20.3)	$19.4 billion (12.5-32.3)	$32.9 billion (21.7-52.5)
*Stockpile, total*	$0.20 billion (0.17-0.24)	$0.22 billion (0.19-0.25)	$0.42 billion (0.36-0.49)

*Note.* All cost in USD 2018.

**Figure 2:**
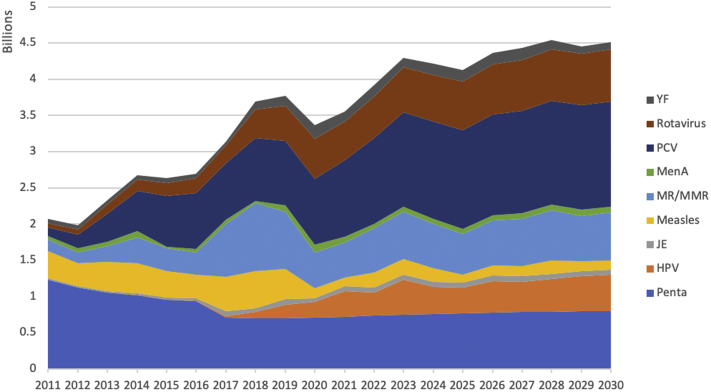
Total immunization program cost by vaccine for 94 countries from 2011 to 2030.

The relative proportion of vaccine and delivery costs of HPV, PCV, and RV vaccines increases over time as more countries introduce these vaccines (Fig. 2). By 2030, 84 countries will have introduced PCV and RV vaccines, and 83 countries will have HPV vaccines in the national immunization schedule by 2030. For HPV, accelerated introduction is anticipated for the next decade since only 37 countries are expected to introduce the vaccine by 2020. Reflecting this trend, the proportion of HPV vaccine and delivery costs to the total cost of immunization programs grows from 0% in 2011 to 11% in 2030, RV, from 3% to 16%, and PCV, from 6% to 32%. A spike in measles outbreaks from 2017 to 2019 led to an increase in the proportion of Measles and MR vaccine and delivery costs to the total cost, amounting 40% in 2018.

Considering the future costs based on the forecasted data for 2018-2030, nine Gavi-supported countries under accelerated transition phase, based on the 2018 transition classification, account for the highest proportion (33.7%) of the total immunization program costs (excluding stockpile costs) of the 94 low- and middle-income countries (Table 3). These countries consist of India, Lao PDR, Nicaragua, Nigeria, Papua New Guinea, Sao Tome and Principe, Solomon Islands, Uzbekistan, and Vietnam, which represent 36.8% of the total number of births from 2018 to 2030.

**Table 3:** Total immunization program costs by Gavi transition classification from 2018 to 2030 (base case scenario).

Gavi transition classification category (2018)	Number of countries	Immunization program costs	Percent of the total cost	Total number of births	Percent of total births
Initial self-financing∗	31	$14.8 billion	28.1%	342.4 million	27.5%
Preparatory transition phase†	16	$10.8 billion	20.4%	230.3 million	18.5%
Accelerated transition phase‡	9	$17.9 billion	33.7%	458.7 million	36.8%
Fully self-financing§	17	$3.9 billion	7.5%	104.1 million	8.4%
Not eligible for Gavi support‖	21	$5.5 billion	10.3%	109.4 million	8.4%
Total (excl. Stockpile)	94	$52.9 billion	100%	1.2 billion	100%

*Note.* All costs in USD 2018.

∗ Initial self-financing: Afghanistan, Benin, Burkina Faso, Burundi, Central African Republic, Chad, Comoros, Democratic Republic of Congo, Eritrea, Ethiopia, Gambia, Guinea, Guinea-Bissau, Haiti, Democratic People’s Republic of Korea, Liberia, Madagascar, Malawi, Mali, Mozambique, Nepal, Niger, Rwanda, Senegal, Sierra Leone, Somalia, South Sudan, United Republic of Tanzania, Togo, Uganda, Zimbabwe.

† Preparatory transition phase: Bangladesh, Cambodia, Cameroon, Cote d’Ivoire, Djibouti, Ghana, Kenya, Kyrgyzstan, Lesotho, Mauritania, Myanmar, Pakistan, Sudan, Tajikistan, Yemen, Zambia.

‡ Accelerated transition phase: India, Lao People’s Democratic Republic, Nicaragua, Nigeria, Papua New Guinea, Sao Tome and Principe, Solomon Islands, Uzbekistan, Viet Nam.

§ Fully self-financing: Angola, Armenia, Azerbaijan, Bhutan, Plurinational State of Bolivia, Congo, Cuba, Georgia, Guyana, Honduras, Indonesia, Kiribati, Republic of Moldova, Mongolia, Sri Lanka, Timor-Leste, Ukraine.

‖ Not eligible for Gavi support: Belize, Cape Verde, Egypt, El Salvador, Fiji, Guatemala, Iraq, Kosovo, Marshall Islands, Federated States of Micronesia, Morocco, Paraguay, Philippines, Samoa, Eswatini, Syrian Arab Republic, Tonga, Turkmenistan, Tuvalu, Vanuatu, West Bank and Gaza.

**Figure 3:**
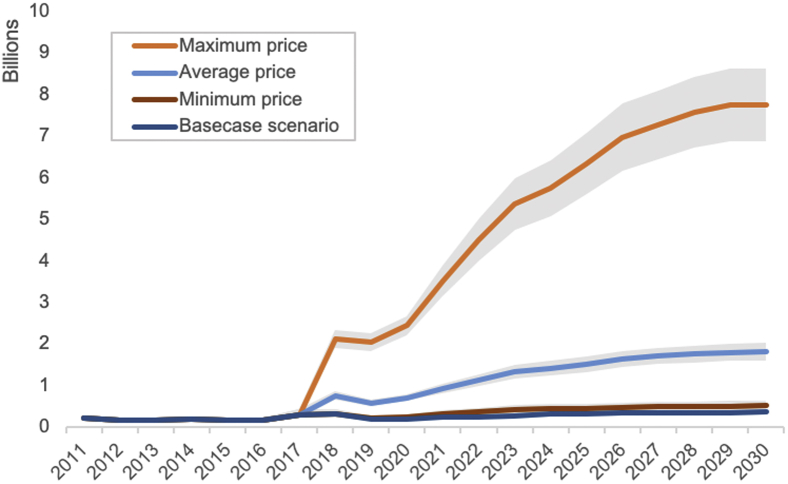
Total immunization program costs for 17 fully self-financing countries from 2018 to 2030, by vaccine price scenario.

Figure 3 shows the results from the alternative price scenarios for 17 fully self-financing countries during 2018-2030. The immunization program costs for these countries from 2018 to 2030 vary significantly across price scenarios, ranging from $6.8 billion ($5.7-$8.6) for the minimum price to $70.9 billion ($62.8-$79.1), compared with the base case scenario, where the costs are 5.4 billion ($4.3–$7.1). Using the average price by antigen and by volume, the immunization program costs for 17 countries will be $18.5 billion ($16.3-$21.1), 244.6% (196.7%-275.0%) higher than the base case scenario.

## Discussion

The results from this study could inform the resource mobilization efforts by the global immunization community for the next decade, such as Gavi’s replenishment, and provide insights on key strategic areas where resources should be deployed to achieve sustainable impact of immunization programs.

The results show an upward trend in the total immunization program costs from 2011 to 2030. Increasing coverage rates in addition to new vaccine introduction, as projected in Gavi’s operational forecast, lead to the increase in the total number of routine doses. Relatively high prices of newer vaccines such as HPV, PCV, and RV vaccines and introduction costs added to the rising total costs over the time horizon.

Delivery costs account for a substantial proportion of the total immunization program costs, and they surpass vaccine costs under the assumption where incremental delivery cost per dose increases with coverage rate. However, limited available evidence in low and lower middle-income countries used to derive the cost function warrants caution in the interpretation of the results from the scenario analysis. More empirical evidence on the relationship between delivery costs and coverage rate for national-level scale-up will be needed for countries and donors to improve health system efficiency.

For HPV, PCV, and RV vaccines, demand–supply balance is critical for realizing the projected introduction schedule outlined in the forecast and sustaining access to these vaccines. Supply constraints led to delays in HPV introduction in recent years, prompting careful monitoring and coordination.^35^ Mitigating supply risks will be particularly important since future country demand is expected to accelerate given the WHO’s call for cervical cancer elimination initiative.^35^ Market-shaping efforts by the global immunization community should be promoted further to enable competitive market conditions and entry of new suppliers, ensuring affordability, security of vaccine supply, and economic viability of suppliers. While positive consequences of these efforts are evidenced by the relative stability of the pentavalent vaccine market,^36^ the exit of one manufacturer in 2017^37^ caused by lower vaccine prices calls for continued monitoring of changing market forces and dynamics.

Countries in the accelerated transition phase account for the highest proportion of immunization program costs excluding stockpile costs from 2018 to 2030. This indicates the importance of transition assessments and plans that provide countries with roadmaps for sustainable financing and implementation of immunization programs. Such roadmaps will require in-depth assessments of country capacity and adequate strategies in areas such as evidence-based decision making, financial sustainability, delivery-related capacity including data management and human resources training, procurement, and national regulatory authorities (NRA), among others.^38^

There is a significant level of uncertainty regarding immunization program costs for fully self-financing countries after they no longer have access to Gavi prices. High vaccine prices could serve as barriers to vaccine access and deteriorate immunization performance in these countries. The results from this study reaffirm the need to develop procurement strategies and capacity for fully self-financing countries as well as their integration into the broader global initiatives targeting middle-income countries.

There are remaining gaps in application of the results of the study as a result of several limitations of the study and the changing landscape of global health and development caused by coronavirus disease 2019 (COVID-19).

First, there is uncertainty regarding the future trend of coverage rates and vaccine introduction dates that can be affected by exogenous factors such as supply constraints, health system capacity, implementation challenges, or global pandemics.

In particular, the COVID-19 pandemic has led to disruption or total suspension of routine childhood immunization services and preventive mass vaccination campaigns.^39^ The continued transmission of COVID-19 will have a substantial impact on future coverage rates and introduction of new vaccines owing to various factors such as limited health workforce capacity and delayed deliveries caused by lockdown measures and the reduced number of flights available for vaccine shipment.^39^

Another limitation is the availability and quality of data for delivery costs. Although we focused only on baseline year expenditure data as opposed to 5-year projection data, accuracy and reliability of country reporting can still affect the quality of cMYP. Furthermore, a significant level of heterogeneity can be found in empirical estimates from the IDCC, especially regarding the quality of studies, composition of cost components, and study settings.

The scope of this study does not cover other vaccines included in national immunization programs, vaccines targeted by the 2018 Vaccine Investment Strategy^40^ that will be supported by Gavi in the next decade, late-stage vaccine candidates that will likely hit the market by 2030, or SARS-CoV-2 vaccines. Additional data on future coverage rate, vaccine prices, vaccine-specific characteristics, and delivery strategies will be needed to calculate the future costs of immunization programs for these vaccines.

Finally, although this study presents the total costs of immunization programs for 10 vaccines, it does not specify the specific bearer of the costs and their burden of financing. Future research is needed to examine the distribution of costs across contributors, the expected level of financing, and the potential funding gap. The Gavi board’s recent decision, as an immediate and interim response to COVID-19, reflects changes in the cofinancing obligations of individual countries.^41^ Because future severe acute respiratory syndrome coronavirus 2 (SARS-CoV-2) vaccines, once available for widespread use, will increase further immunization program costs and the burden of financing, more in-depth analyses will be needed to provide additional resources and support for new vaccine deployment in LMICs. Nevertheless, to our knowledge, this is the most up-to-date analysis of immunization program costs at the global level, leveraging the latest available data on model parameters. Although this study does not capture the impact of COVID-19 on the costs of immunization programs against vaccine preventable diseases, the “business as usual” scenario presented in the study will inform mobilization and safeguarding of resources needed to resume and continue immunization programs and facilitate longer-term transition planning at the global level.

## Conclusion

The results of this study demonstrate an upward trend of immunization program costs from 2011 to 2030 with varying assumptions about immunization delivery cost. Resource mobilization efforts at the global and country levels will be needed to reach the level of investment needed for the coming decade. Global-level initiatives, vaccine price reductions, and targeted strategies for transitioning countries will help ensure the sustainability of immunization programs, especially given the ongoing challenges of COVID-19.

## Supplemental Material

Appendices 1-8
