# Supplementary material for: Costs of Immunization Programs for 10 Vaccines in 94 Low- and Middle-Income Countries From 2011 to 2030
Source: Value Health. 2021 Jan;24(1):70–7. doi: 10.1016/j.jval.2020.07.010 (PMC7813215; doi:10.1016/j.jval.2020.07.010)
Supplement: Appendices 1-8 [file mmc1.zip › jval_3053_mmc1.dotx]

**Supplementary materials**

Appendix 1. Full list of countries

| **No** | **Country** | **WHO Region** | **Gavi eligible** | **World Bank Income Group** | **Gavi Transition classification** |
| --- | --- | --- | --- | --- | --- |
|  |  |  |  | **2018** | **2018** |
| 1 | Afghanistan | EMRO | Gavi | Low-income | Initial self-financing |
| 2 | Angola | AFRO | Gavi | Lower-middle income | Fully self-financing |
| 3 | Armenia | EURO | Gavi | Upper-middle income | Fully self-financing |
| 4 | Azerbaijan | EURO | Gavi | Upper-middle income | Fully self-financing |
| 5 | Bangladesh | SEARO | Gavi | Lower-middle income | Preparatory transition phase |
| 6 | Belize | AMRO | PAHO | Upper-middle income | Not eligible |
| 7 | Benin | AFRO | Gavi | Low-income | Initial self-financing |
| 8 | Bhutan | SEARO | Gavi | Lower-middle income | Fully self-financing |
| 9 | Bolivia | AMRO | Gavi | Lower-middle income | Fully self-financing |
| 10 | Burkina Faso | AFRO | Gavi | Low-income | Initial self-financing |
| 11 | Burundi | AFRO | Gavi | Low-income | Initial self-financing |
| 12 | Cambodia | WPRO | Gavi | Lower-middle income | Preparatory transition phase |
| 13 | Cameroon | AFRO | Gavi | Lower-middle income | Preparatory transition phase |
| 14 | Cape Verde | AFRO | Non | Lower-middle income | Not eligible |
| 15 | Central African Republic | AFRO | Gavi | Low-income | Initial self-financing |
| 16 | Chad | AFRO | Gavi | Low-income | Initial self-financing |
| 17 | Comoros | AFRO | Gavi | Low-income | Initial self-financing |
| 18 | Congo, Dem. Rep. | AFRO | Gavi | Low-income | Initial self-financing |
| 19 | Congo | AFRO | Gavi | Lower-middle income | Fully self-financing |
| 20 | Cote d'Ivoire | AFRO | Gavi | Lower-middle income | Preparatory transition phase |
| 21 | Cuba | AMRO | Gavi | Upper-middle income | Fully self-financing |
| 22 | Djibouti | EMRO | Gavi | Lower-middle income | Preparatory transition phase |
| 23 | Egypt | EMRO | Non | Lower-middle income | Not eligible |
| 24 | El Salvador | AMRO | PAHO | Lower-middle income | Not eligible |
| 25 | Eritrea | AFRO | Gavi | Low-income | Initial self-financing |
| 26 | Ethiopia | AFRO | Gavi | Low-income | Initial self-financing |
| 27 | Fiji | WPRO | Non | Upper-middle income | Not eligible |
| 28 | Gambia | AFRO | Gavi | Low-income | Initial self-financing |
| 29 | Georgia | EURO | Gavi | Lower-middle income | Fully self-financing |
| 30 | Ghana | AFRO | Gavi | Lower-middle income | Preparatory transition phase |
| 31 | Guatemala | AMRO | PAHO | Upper-middle income | Not eligible |
| 32 | Guinea | AFRO | Gavi | Low-income | Initial self-financing |
| 33 | Guinea-Bissau | AFRO | Gavi | Low-income | Initial self-financing |
| 34 | Guyana | AMRO | Gavi | Upper-middle income | Fully self-financing |
| 35 | Haiti | AMRO | Gavi | Low-income | Initial self-financing |
| 36 | Honduras | AMRO | Gavi | Lower-middle income | Fully self-financing |
| 37 | India | SEARO | Gavi | Lower-middle income | Accelerated transition phase |
| 38 | Indonesia | SEARO | Gavi | Lower-middle income | Fully self-financing |
| 39 | Iraq | EMRO | Non | Upper-middle income | Not eligible |
| 40 | Kenya | AFRO | Gavi | Lower-middle income | Preparatory transition phase |
| 41 | Kiribati | WPRO | Gavi | Lower-middle income | Fully self-financing |
| 42 | Korea, DPR | SEARO | Gavi | Low-income | Initial self-financing |
| 43 | Kosovo | EURO | Non | Lower-middle income | Not eligible |
| 44 | Kyrgyzstan | EURO | Gavi | Lower-middle income | Preparatory transition phase |
| 45 | Lao PDR | WPRO | Gavi | Lower-middle income | Accelerated transition phase |
| 46 | Lesotho | AFRO | Gavi | Lower-middle income | Preparatory transition phase |
| 47 | Liberia | AFRO | Gavi | Low-income | Initial self-financing |
| 48 | Madagascar | AFRO | Gavi | Low-income | Initial self-financing |
| 49 | Malawi | AFRO | Gavi | Low-income | Initial self-financing |
| 50 | Mali | AFRO | Gavi | Low-income | Initial self-financing |
| 51 | Marshall Islands | WPRO | Non | Upper-middle income | Not eligible |
| 52 | Mauritania | AFRO | Gavi | Lower-middle income | Preparatory transition phase |
| 53 | Micronesia | WPRO | Non | Lower-middle income | Not eligible |
| 54 | Moldova | EURO | Gavi | Lower-middle income | Fully self-financing |
| 55 | Mongolia | WPRO | Gavi | Lower-middle income | Fully self-financing |
| 56 | Morocco | EMRO | Non | Lower-middle income | Not eligible |
| 57 | Mozambique | AFRO | Gavi | Low-income | Initial self-financing |
| 58 | Myanmar | SEARO | Gavi | Lower-middle income | Preparatory transition phase |
| 59 | Nepal | SEARO | Gavi | Low-income | Initial self-financing |
| 60 | Nicaragua | AMRO | Gavi | Lower-middle income | Accelerated transition phase |
| 61 | Niger | AFRO | Gavi | Low-income | Initial self-financing |
| 62 | Nigeria | AFRO | Gavi | Lower-middle income | Accelerated transition phase |
| 63 | Pakistan | EMRO | Gavi | Lower-middle income | Preparatory transition phase |
| 64 | Papua New Guinea | WPRO | Gavi | Lower-middle income | Accelerated transition phase |
| 65 | Paraguay | AMRO | PAHO | Upper-middle income | Not eligible |
| 66 | Philippines | WPRO | Non | Lower-middle income | Not eligible |
| 67 | Rwanda | AFRO | Gavi | Low-income | Initial self-financing |
| 68 | Samoa | WPRO | Non | Upper-middle income | Not eligible |
| 69 | Sao Tome and Principe | AFRO | Gavi | Lower-middle income | Accelerated transition phase |
| 70 | Senegal | AFRO | Gavi | Low-income | Initial self-financing |
| 71 | Sierra Leone | AFRO | Gavi | Low-income | Initial self-financing |
| 72 | Solomon Islands | WPRO | Gavi | Lower-middle income | Accelerated transition phase |
| 73 | Somalia | EMRO | Gavi | Low-income | Initial self-financing |
| 74 | Sri Lanka | SEARO | Gavi | Lower-middle income | Fully self-financing |
| 75 | Sudan: North | EMRO | Gavi | Lower-middle income | Preparatory transition phase |
| 76 | Sudan: South | AFRO | Gavi | Low-income | Preparatory transition phase |
| 77 | Eswatini | AFRO | Non | Lower-middle income | Not eligible |
| 78 | Syria | EMRO | Non | Low-income | Not eligible |
| 79 | Tajikistan | EURO | Gavi | Low-income | Preparatory transition phase |
| 80 | Tanzania | AFRO | Gavi | Low-income | Initial self-financing |
| 81 | Timor-Leste | SEARO | Gavi | Lower-middle income | Fully self-financing |
| 82 | Togo | AFRO | Gavi | Low-income | Initial self-financing |
| 83 | Tonga | WPRO | Non | Upper-middle income | Not eligible |
| 84 | Turkmenistan | EURO | Non | Upper-middle income | Not eligible |
| 85 | Tuvalu | WPRO | Non | Upper-middle income | Not eligible |
| 86 | Uganda | AFRO | Gavi | Low-income | Initial self-financing |
| 87 | Ukraine | EURO | Gavi | Lower-middle income | Fully self-financing |
| 88 | Uzbekistan | EURO | Gavi | Lower-middle income | Accelerated transition phase |
| 89 | Vanuatu | WPRO | Non | Lower-middle income | Not eligible |
| 90 | Viet Nam | WPRO | Gavi | Lower-middle income | Accelerated transition phase |
| 91 | West Bank and Gaza | EMRO | Non | Lower-middle income | Not eligible |
| 92 | Yemen | EMRO | Gavi | Low-income | Preparatory transition phase |
| 93 | Zambia | AFRO | Gavi | LMIC | Preparatory transition phase |
| 94 | Zimbabwe | AFRO | Gavi | Low-income | Initial self-financing |

Appendix 2. Scope of the analysis

|  | **Vaccines** | **Strategy** | **RI*** | **SIA**** | **Stockpile** |
| --- | --- | --- | --- | --- | --- |
| 1 | Pentavalent | Infants (3 doses) | ✓ |  |  |
| 2 | Human papillomavirus | Girls age 10;  Multi-age cohort (2 doses) | ✓ | ✓ |  |
| 3 | Japanese encephalitis | Infants (1 dose);  Campaign (1 dose) | ✓ | ✓ |  |
| 4 | Measles | Infants (1^st^ and 2^nd^);  Campaign (1 dose) | ✓ | ✓ |  |
| 5 | Measles-Rubella (MR) | Infants (1^st^ and 2^nd^);  Campaign (1 dose) | ✓ | ✓ |  |
| 6 | Measles- Mumps-Rubella (MMR) | Infants (1^st^ and 2^nd^);  Campaign (1 dose) | ✓ | ✓ |  |
| 7 | Meningococcal group A conjugate (MenA) | Infants (1 dose)  Campaign (1 dose) | ✓ | ✓ | ✓ |
| 8 | Pneumococcal conjugate | Infants (3 doses) | ✓ |  |  |
| 9 | Rotavirus | Infants (2 or 3 doses) | ✓ |  |  |
| 10 | Yellow fever | Infants (1 dose)  Campaign (1 dose) | ✓ | ✓ | ✓ |

Appendix 3. Immunization delivery cost components: comparison between cMYPs and IDCC

| **Category** | **cMYPs^a^** | **IDCC^b^** |
| --- | --- | --- |
| Labor  Function | Personnel - Salaries of full-time NIP health workers (immunization specific) | Paid human resources |
|  | Personnel - Per diems for outreach vaccinators/mobile teams Per diems for supervision and monitoring | Per diem and travel allowances |
|  | No corresponding category | Volunteer human resources |
|  | Shared personnel costs | No corresponding category |
| Storage  Function | Cold chain equipment  Cold chain maintenance and overhead | Cold chain equipment and their overheads (installation, energy, maintenance, repairs) |
| Transportation function | Vehicles  Transportation for fixed site strategy (incl. vaccine distribution)  Transportation for outreach strategy  Transportation for mobile strategy  Shared transportation costs | Vehicles, transport and fuel |
| Other capital | Other capital equipment (under routine capital costs)  Maintenance of other capital equipment  Building construction  Building overheads (electricity, water,..etc) | Buildings, utilities, other overheads and/or shared costs |
| Other recurrent | Program management | Program management |
|  | Short-term training | Training and capacity building |
|  | IEC/social mobilization | Social mobilization and advocacy |
|  | Disease surveillance | AEFI and disease surveillance |
|  | No corresponding category | Wastage management |
|  | Other routine recurrent costs | Other supplies and recurrent costs |

^a^ Comprehensive Multi-Year Plans (cMYP)

^b^ Immunization Delivery Cost Catalogue (IDCC)

Appendix 4. List of predictors and data sources

| **No** | **Indicators** | **Source** |
| --- | --- | --- |
| 1 | Land area (sq.km)* | World Bank Open Data |
| 2 | Maternal mortality ratio (per 100,000 live births) | World Bank Open Data |
| 3 | Under 5 mortality rate (per 1000 live births) | UN World Population Prospects (VIMC) |
| 4 | Total number of births | UN World Population Prospects (VIMC) |
| 5 | Pregnant women receiving prenatal care* | World Bank Open Data |
| 6 | Population growth (annual %) | UN World Population Prospects (VIMC) |
| 7 | Total population | World Bank Open Data |
| 8 | Population density (people per sq.km of land area) | World Bank Open Data |
| 9 | Poverty headcount ratio at $1.90 a day (2011 PPP) (%)* | World Bank Open Data |
| 10 | Urban population (% of total population)* | World Bank Open Data |
| 11 | GDP growth (annual %) | World Bank Open Data |
| 12 | Access to electricity (% of population) | World Bank Open Data |
| 13 | Investment in transport with private participation (current US$) | World Bank Open Data |
| 14 | Electric power consumption (kWh per capita) | World Bank Open Data |
| 15 | GDP per capita* | World Bank Open Data |
| 16 | Domestic general government health expenditure as % GDP | WHO NHA database |
| 17 | DTP3 coverage rate (%) | UN World Population Prospects (VIMC) |
| 18 | Total number of DTP3 doses delivered* | DOVE team calculation  based on VIMC data |
| 19 | Total number of doses* | DOVE team calculation  based on VIMC data |

*Predictors identified from cost determinant studies

Appendix 5. Results from k-fold cross-validation (k=10)

|  | **Model** | **Data** | **1** | **2** | **3** | **4** | **5** | **6** | **7** | **8** | **9** | **10** | **Average RMSE** |
| --- | --- | --- | --- | --- | --- | --- | --- | --- | --- | --- | --- | --- | --- |
| 1 | Combined model | Combined dataset | 1.404 | 1.159 | 0.969 | 1.110 | 0.911 | 1.235 | 1.232 | 1.219 | 0.963 | 1.099 | 1.130 |
| 2 | Combined model | cMYP dataset | 1.003 | 1.102 | 1.065 | 0.832 | 1.033 | 1.288 | 0.820 | 0.811 | 0.940 | 0.805 | 0.970 |
| 3 | Combined model | IDCC dataset | 1.076 | 1.213 | 0.597 | 1.424 | 1.386 | 0.844 | 1.089 | 0.747 | 1.965 | 0.806 | 1.115 |
| 4 | cMYP model | Combined dataset | 0.671 | 1.441 | 0.857 | 1.005 | 1.032 | 1.566 | 1.457 | 0.950 | 1.406 | 0.860 | 1.125 |
| 5 | cMYP model | cMYP dataset | 1.242 | 1.060 | 1.082 | 0.889 | 0.812 | 1.131 | 0.559 | 1.265 | 1.029 | 0.833 | 0.990 |
| 6 | cMYP model | IDCC dataset | 1.157 | 0.951 | 1.267 | 1.369 | 0.884 | 1.664 | 2.051 | 1.104 | 0.829 | 0.662 | 1.194 |
| 7 | IDCC model | Combined dataset | 1.269 | 0.784 | 1.122 | 1.270 | 1.035 | 0.864 | 1.263 | 1.159 | 0.980 | 1.297 | 1.104 |
| 8 | IDCC model | cMYP dataset | 1.129 | 0.798 | 1.146 | 0.948 | 0.322 | 3.112 | 1.353 | 1.375 | 1.045 | 1.150 | 1.238 |
| 9 | IDCC model | IDCC dataset | 1.883 | 1.325 | 1.095 | 0.958 | 1.354 | 0.920 | 1.281 | 1.129 | 0.903 | 0.943 | 1.179 |

| **Performance** | **Average RMSE** |
| --- | --- |
| cMYP model | 1.103 |
| IDCC model | 1.174 |
| **Combined model** | **1.072** |

Each data set was randomly partitioned into k equal sized subsamples (k=10 in our analysis). A single subsample as the validation (“test”) data and (k-1) subsamples were used as training data. We conducted out-of-sample validation for nine scenarios and compared relative performance by model and by dataset measured based on the average root mean square error.

Appendix 6. Results of multiple linear regression

5.1. Multiple linear regression using the final model

$${Cost per dose}_{j}=Exp [ \beta_{0}+ \beta_{1}X_{1}+\beta_{2}X_{2}+\beta_{3}X_{3}+\beta_{4}X_{4}+\beta_{5}X_{5}+\beta_{6}X_{6}+\beta_{7}X_{7}+\beta_{8}X_{8}+\beta_{9}X_{9}+ \beta_{10}X_{10}+ \beta_{11}X_{11}+\sum j\frac{e_{j}}{N}]$$

$X_{1}= Urban population$ *(% of total population)*

$X_{2}=Total number of births$

$X_{3}=GDP growth rate$ *(% annual)*

$X_{4}=DTP3 coverage rate$ *(%)*

$X_{5}=GDP per capita$ *(current US$)*

$X_{6}=Land area$ *(sq.km)*

$X_{7}=Population growth rate$ *(% annual)*

$X_{8}= Total number of doses delivered$

$X_{9}=Maternal mortality ratio$ *(per 100,000 live births)*

$X_{10}=Total number of DTP3 doses delivered$

$X_{11}=Population density$ *(people per sq.km of land area)*

$e_{j}=Random Error for country j$

| Variable | Coefficient | Standard Error | P-Value | 95% Confidence Interval |
| --- | --- | --- | --- | --- |
| Urban | -.0023195 | .0174127 | 0.894 | (-0.0367, 0.032) |
| Birth | 5.52e-07 | 2.71e-07 | 0.043 | (1.76e-08, 1.09e-06) |
| GDP growth | -.0258438 | .0439864 | 0.558 | (-.1127169, .0610293) |
| Dtp3 coverage | 1.743651 | 2.006666 | 0.386 | (-2.219508, - 5.706809) |
| GDP per capita | -.0000196 | .0002714 | 0.942 | (-.0005557, .0005164) |
| Land area | -4.26e-07 | 5.85e-07 | 0.468 | (-1.58e-06, 7.30e-07) |
| Population growth | -.4109075 | .2516257 | 0.104 | (-.9078674, .0860523) |
| Total number of doses | -2.87e-08 | 1.57e-08 | 0.069 | (-5.97e-08, 2.23e-09) |
| Maternal mortality ratio | -.0014653 | .0013122 | 0.266 | (-.0040569, .0011262) |
| DTP3 doses | -3.34e-07 | 4.26e-07 | 0.434 | (-1.18e-06, 5.07e-07) |
| Population density | -.0002507 | .0015785 | 0.874 | (-.0033682, .0028669) |
| Constant | 2.469589 | 2.138158 | 0.250 | (-1.753264, 6.692442) |

5.2. Adding a dummy variable of cMYP

| Variable | Coefficient | Standard Error | P-Value | 95% Confidence Interval |
| --- | --- | --- | --- | --- |
| Dummy (cMYP=1, IDCC=0) | .6597162 | .6012096 | 0.274 | (-.5277281, 1.84716) |
| Urban | -.000848 | .0174531 | 0.961 | (-.0353194, .0336234) |
| Birth | 7.34e-07 | 3.17e-07 | 0.022 | (1.08e-07, 1.36e-06) |
| GDP growth | -.014429 | .0451723 | 0.750 | (-.1036485, .0747905) |
| Dtp3 coverage | 2.036023 | 2.023003 | 0.316 | (-1.959594, 6.031639) |
| GDP per capita | -.0000438 | .0002721 | 0.872 | (-.0005813,.0004937) |
| Land area | -5.28e-07 | 5.92e-07 | 0.374 | (-1.70e-06,6.42e-07 ) |
| Population growth | -.4260995 | .2518452 | 0.093 | (-.9235169, .071318 ) |
| Total number of doses | -4.43e-08 | 2.11e-08 | 0.038 | (-8.60e-08, -2.56e-09) |
| Maternal mortality ratio | -.0014768 | .0013114 | 0.262 | (-.0040669 , .0011134) |
| DTP3 doses | -2.16e-07 | 4.39e-07 | 0.624 | (-1.08e-06, 6.52e-07) |
| Population density | -.0000222 | .0015912 | 0.989 | (-.0031649, .0031206) |
| Constant | 1.757413 | 2.233177 | 0.432 | (-2.653317, 6.168144 ) |

Appendix 7. Methodology for scenario analysis: cost-function to estimate the intervention cost per percent increase in coverage rate

Summary of log-linear regression model (Ozawa et al. 2018)

|  | $\beta$ | Exp ($\beta)$ | S.E. | p-value |
| --- | --- | --- | --- | --- |
| Baseline coverage | 0.0357 | 1.0363 | 0.0107 | 0.001 |
| Relative high income^a^ | 1.5040 | 4.4999 | 2.7073 | 0.012 |
| SIA delivery | 0.4014 | 1.4939 | 1.0168 | 0.555 |
| Constant | -3.5201 | 0.0295 | 0.0214 | 0.000 |
| RMSE = 1.7576 |  |  |  |  |
| R^2^=0,3134 |  |  |  |  |

Source: Ozawa et al. 2018

^a^ Relatively low-income countries as a reference group

^b^ Routine delivery cost as a reference group

We predicted cost per dose estimate for the coverage rate for each year for each country using the log-linear model above. We then multiplied the cost per dose by the number of doses for the corresponding year.

Appendix 8. Scenario analysis for prices

Market Information for Access to Vaccines (MI4A) database (formerly V3P) contains vaccine purchase (product, price and procurement) data as reported by countries via the WHO/UNICEF Joint Reporting Form (JRF).

We only selected those data from 2011- 2018 and are under alternative procurement mechanism which is defined as a combination of self-procurement, other, other pool procurement and sub-regional pool procurement. Those data under UNICEF SD and PAHO RF procurement mechanism were excluded.

Under the selected data, we defined purchasing volume of each antigen into three categories - Low, Medium, and High – using the calculated interquartile range (IQR) of annual number of doses as a parameter. All data in Q1 (below 25%) are defined as “Low”, Q2 (between 25%-75%) are defined as “Medium”, and Q3 (more than 75%) are defined as “High”. Table 7.1. shows the volume distribution of 10 vaccines. We defined the price range for each antigen (Table 2) by identifying the minimum price, maximum price and average price in each volume category. All prices are in 2018 USD.

*Note: MI4A contains no data under self-procurement mechanism for Meningitis A vaccine, only UNICEF SD and PAHO RF procurement data. For MenA, we assumed that the volume falls under “Medium” category and applied the average % price difference (875%) between the MI4A price and Gavi price for “Medium” volume across all vaccines to project future MenA price under alternative procurement.

7.1. Volume category for each vaccine

| **Vaccine** | **Low** | **Medium** | **High** |
| --- | --- | --- | --- |
| **DTwP-HepB-Hib** | 3,000-120,437 | 120,437-2,400,000 | 2,400,000-87,475,000 |
| **HPV** | 21-4,569.5 | 4,569.5-216,362.5 | 216,362.5-14,000,000 |
| **JE** | 500-211,860 | 211,860-1,350,000 | 1350,000-56,000,000 |
| **Measles** | 300-129,600 | 129,600-2,700,000 | 2,700,000-41,900,000 |
| **MenA conj.** | N/A | N/A | N/A |
| **MMR** | 40-2,9560.25 | 29,560.25-449,917.5 | 449,917.5-33,400,000 |
| **MR** | 6,000-196,000 | 196,000-3,282,482.5 | 3,282,482.5-216,500,000 |
| **PCV** | 40-11,509.5 | 11,509.5-573,500 | 573,500-21,000,000 |
| **Rota** | 600-40,000 | 40,000-1,800,000 | 1,800,000-33,850,000 |
| **YF** | 50-974.25 | 974.25-12,277 | 12,277-42,000,000 |

7.2. Minimum, maximum and average price for each volume category for each vaccine

| Vaccine | Volume | Min | Max | Average |
| --- | --- | --- | --- | --- |
| DTwP-HepB-Hib | **Low** | $ 0.58 | $ 6.18 | $ 3.87 |
| DTwP-HepB-Hib | **Medium** | $ 0.30 | $ 6.45 | $ 2.87 |
| DTwP-HepB-Hib | **High** | $ 0.45 | $ 6.60 | $ 2.31 |
| HPV | **Low** | $ 18.51 | $ 168.10 | $ 65.04 |
| HPV | **Medium** | $ 9.52 | $ 144.18 | $ 41.81 |
| HPV | **High** | $ 6.09 | $ 168.10 | $ 35.69 |
| JE | **Low** | $ 3.50 | $ 37.27 | $ 18.02 |
| JE | **Medium** | $ 0.44 | $ 11.14 | $ 5.71 |
| JE | **High** | $ 0.41 | $ 7.65 | $ 1.21 |
| Measles | **Low** | $ 2.57 | $ 6.61 | $ 4.14 |
| Measles | **Medium** | $ 0.21 | $ 3.90 | $ 0.98 |
| Measles | **High** | $ 0.16 | $ 2.20 | $ 0.59 |
| MMR | **Low** | $ 1.81 | $ 21.05 | $ 8.84 |
| MMR | **Medium** | $ 0.63 | $ 43.79 | $ 7.75 |
| MMR | **High** | $ 1.22 | $ 21.09 | $ 5.83 |
| MR | **Low** | $ 0.62 | $ 3.60 | $ 2.42 |
| MR | **Medium** | $ 0.62 | $ 10.28 | $ 1.54 |
| MR | **High** | $ 0.55 | $ 10.91 | $ 1.89 |
| PCV | **Low** | $ 9.41 | $ 131.77 | $ 53.14 |
| PCV | **Medium** | $ 14.93 | $ 114.34 | $ 37.39 |
| PCV | **High** | $ 7.04 | $ 130.07 | $ 27.49 |
| Rota | **Low** | $ 9.66 | $ 75.98 | $ 25.86 |
| Rota | **Medium** | $ 4.75 | $ 93.27 | $ 22.58 |
| Rota | **High** | $ 0.75 | $ 70.81 | $ 12.51 |
| YF | **Low** | $ 20.04 | $ 87.84 | $ 41.55 |
| YF | **Medium** | $ 2.10 | $ 61.47 | $ 21.40 |
| YF | **High** | $ 1.08 | $ 23.49 | $ 11.39 |
| MenA | **Medium** | $ 0.49 | $ 25.70 | $ 5.50 |
